# Effectiveness of adding motivational interviewing or a stratified vocational advice intervention to usual case management on return to work for people with musculoskeletal disorders: the MI-NAV randomised controlled trial

**DOI:** 10.1136/oemed-2022-108637

**Published:** 2022-11-25

**Authors:** Fiona Aanesen, Margreth Grotle, Tarjei Langseth Rysstad, Anne Therese Tveter, Alexander Tingulstad, Ida Løchting, Milada C Småstuen, Maurits W van Tulder, Rigmor Berg, Nadine E Foster, Gwenllian Wynne-Jones, Gail Sowden, Egil Fors, Gunnhild Bagøien, Roger Hagen, Kjersti Storheim, Britt Elin Øiestad

**Affiliations:** 1 Department of Rehabilitation Science and Health Technology, Oslo Metropolitan University, Oslo, Norway; 2 Centre for Intelligent Musculoskeletal Health, Department of Rehabilitation Science and Health Technology, Oslo Metropolitan University, Oslo, Norway; 3 Research and Communication Unit for MSK Health (FORMI), Division of Clinical Neuroscience, Oslo University Hospital, Oslo, Norway; 4 Norwegian National Advisory Unit on Rehabilitation in Rheumatology, Diakonhjemmet Hospital, Oslo, Norway; 5 Department of Nursing and Health Promotion, Oslo Metropolitan University, Oslo, Norway; 6 Faculty Behavioural and Movement Sciences, Vrije Universiteit Amsterdam, Amsterdam, The Netherlands; 7 Reviews and Health Technology Assessments, Norwegian Institute of Public Health, Oslo, Norway; 8 Department of Community Medicine, University of Tromsø, Tromsø, Norway; 9 STARS Education and Research Alliance, Surgical Treatment and Rehabilitation Service (STARS), The University of Queensland and Metro North Health, Brisbane, Queensland, Australia; 10 School of Medicine, Keele University, Keele, UK; 11 Connect Health, Newcastle upon Tyne, UK; 12 General Practice Research Unit, Department of Public Health and Nursing, Norwegian University of Science and Technology, Trondheim, Norway; 13 Nidelv Community Mental Health Center, Tiller, Clinic of Mental Health, St Olavs University Hospital, Trondheim, Norway; 14 Department of Psychology, University of Oslo, Oslo, Norway; 15 Department of Psychology, Norwegian University of Science and Technology, Trondheim, Norway; 16 Modum Bad, Research Institute, Vikersund, Norway

**Keywords:** Occupational Health, Rehabilitation, Sick Leave, Musculoskeletal System

## Abstract

**Objectives:**

To evaluate if adding motivational interviewing (MI) or a stratified vocational advice intervention (SVAI) to usual case management (UC), reduced sickness absence over 6 months for workers on sick leave due to musculoskeletal disorders.

**Methods:**

We conducted a three-arm parallel pragmatic randomised controlled trial including 514 employed workers (57% women, median age 49 (range 24–66)), on sick leave for at least 50% of their contracted work hours for ≥7 weeks. All participants received UC. In addition, those randomised to UC+MI were offered two MI sessions from social insurance caseworkers and those randomised to UC+SVAI were offered vocational advice from physiotherapists (participants with low/medium-risk for long-term sickness absence were offered one to two sessions, and those with high-risk were offered three to four sessions).

**Results:**

Median sickness absence was 62 days, (95% CI 52 to 71) in the UC arm (n=171), 56 days (95% CI 43 to 70) in the UC+MI arm (n=169) and 49 days (95% CI 38 to 60) in the UC+SVAI arm (n=169). After adjusting for predefined potential confounding factors, the results showed seven fewer days in the UC+MI arm (95% CI −15 to 2) and the UC+SVAI arm (95% CI −16 to 1), compared with the UC arm. The adjusted differences were not statistically significant.

**Conclusions:**

The MI-NAV trial did not show effect on return to work of adding MI or SVAI to UC. The reduction in sickness absence over 6 months was smaller than anticipated, and uncertain due to wide CIs.

**Trial registration number:**

NCT03871712.

**WHAT IS ALREADY KNOWN ON THIS TOPIC**

- Two previous trials have tested the effect of motivational interviewing (MI), to facilitate return to work (RTW), for people with musculoskeletal disorders, with conflicting results.
- One previous trial has shown that a low intensity vocational advice intervention, reduced sickness absence by 5 days over 4 months for workers with musculoskeletal disorders in the UK.

**WHAT THIS STUDY ADDS**

- The MI-NAV trial showed that adding MI or a stratified vocational advice intervention (SVAI) to usual case management resulted in a non-statistically significant reduction in sickness absence over 6 months for workers on sick leave due to musculoskeletal disorders in Norway.

**HOW THIS STUDY MIGHT AFFECT RESEARCH, PRACTICE OR POLICY**

- The MI and SVAI interventions should be replicated in future trials, powered to detect smaller differences between groups. Prior to conducting new trials, a minimal important difference for RTW outcomes should be decided through involvement of patients and other stakeholders.

## Introduction

Musculoskeletal disorders are the main contributors to years lived with disability worldwide.^1^ In Norway, musculoskeletal disorders are the main cause of sick leave,^2^ and are associated with a significant burden on individuals and economic costs to society.^3^ Work disability and sick leave are influenced by healthcare, individual, social and work-related factors.^4^ To address the large burden related to sick leave, effective individually-tailored interventions targeting barriers to return to work (RTW) are needed.^5^

One intervention recommended in vocational rehabilitation is motivational interviewing (MI).^6^ MI is a person-centred counselling style aimed at increasing motivation for change.^7^ MI has been successful in increasing treatment adherence for people with musculoskeletal disorders^8^ and chronic pain conditions,^9^ and can be effective when provided as a brief intervention.^10^ However, there is sparse evidence on the effectiveness of MI to facilitate RTW.^11 12^

Another intervention to help workers with musculoskeletal disorders to RTW, was developed and tested in the Study of Work And Pain (SWAP) trial in the UK.^13^ The vocational advice intervention was based on the principles of case management to help participants overcome obstacles to RTW.^13^ The SWAP intervention was offered to patients with musculoskeletal disorders consulting in general practices, who were struggling at work or on sick leave for less than 6 months.

Providing interventions to all workers on sick leave is extremely resource demanding, and may not be justified in a Norwegian context given that approximately 80% of the workers RTW during the first 8 weeks of sick leave.^2^ The optimal time window for providing vocational interventions for people with musculoskeletal disorders seems to be between weeks 8 and 12 of sick leave.^14^

It is not known if the SWAP intervention could be effective when delivered as a stratified intervention, tailored according to risk for long-term sickness absence. Therefore, we aimed to assess if adding either MI or a stratified vocational advice intervention (SVAI) to usual case management (UC) reduced sickness absence days over 6 months, for workers with musculoskeletal disorders on sick leave for more than seven consecutive weeks. We conducted two independent comparisons:

1. UC compared with UC+MI.
2. UC compared with UC+SVAI.

## Method

### Design

The MI-NAV trial was a three-arm, pragmatic randomised controlled trial (RCT) with 6 months follow-up, including an internal pilot. We conducted the trial in cooperation with the Norwegian Labour and Welfare Administration (NAV). The methods have been reported previously in the study protocol,^15^ in the process evaluation of the SVAI,^16^ and in the fidelity evaluation of the MI intervention.^17^ The Norwegian Centre for Research Data approved the project (861249), and the trial was conducted in accordance with the Helsinki declaration and the General Data Protection Regulation (GDPR). The trial is reported according to the Consolidated Standards of Reporting Trials extension statement for reporting multi-arm trials,^18^ and CONSORT and SPIRIT Extension for RCTs Revised in Extenuating Circumstanses, (CONSERVE).^19^

### Participants

Participants were workers aged 18–67 years, employed full-time or part-time, on sick leave with musculoskeletal disorders for at least 50% of their contracted work hours for at least seven consecutive weeks. We included workers diagnosed with musculoskeletal disorders listed in the second edition of the International Classification of Primary Care (ICPC-2).^20^ We excluded: those with serious somatic or mental health disorders affecting their work ability and in need of specialised treatment (eg, cancer, psychotic disorders), pregnant women, unemployed, freelancers and self-employed workers and those lacking sufficient Norwegian or English language skills to answer the questionnaires or communicate by telephone.

### Recruitment, stratification and randomisation

From April 2019 to October 2020 workers on sick leave due to musculoskeletal disorders were phoned from the NAV directorate. Every week the recruiters received lists of workers in week seven of sick leave, affiliated to eight NAV offices in South-Eastern Norway. Eligible candidates were informed about the trial and assured that participation was voluntary and did not affect sick leave benefits or UC provided by the NAV. Workers who agreed to participate received an electronic link to written information about the trial, an electronic informed consent form and the baseline questionnaire.

We used the Örebro Musculoskeletal Pain Screening Questionnaire Short Form (ÖMPSQ-SF),^21^ and the Keele STarT MSK Tool,^22 23^ to stratify the participants into two risk groups of long-term sick leave (described in online supplemental appendix 1). Participants with ≥9 on the Keele STarT MSK Tool and ≥60 on the ÖMPSQ-SF were stratified to a ‘high-risk group’, all others were stratified to a ‘medium/low-risk group’. After stratification to the risk-group, participants were randomly allocated (1:1:1 allocation within each stratum of low/medium and high-risk). Group allocation was concealed for the recruitment staff. A statistician (MCS), with no involvement in the running of the trial, prepared a computer-generated allocation sequence for each risk-group, only available for the person in charge of group allocation (TLR).

10.1136/oemed-2022-108637.supp1 Supplementary data

### Interventions

The interventions are described in detail in online supplemental appendix 1, and in the published fidelity and process evaluation.^16 17^ All participants were offered UC for people on sick leave in Norway. In Norway, workers on sick leave are entitled to full wage replacement benefits for up to 12 months. The first 16 days are covered by the employer, the rest by the social security system administered through the NAV. In addition, participants randomised to the UC+MI arm were offered two face-to-face sessions of MI from a NAV caseworker. The first session was delivered at a local NAV office as soon as possible after inclusion, and the second session was held 2 weeks later. The participants in the UC+SVAI arm were offered vocational advice and case management from physiotherapists. Those stratified to the low/medium-risk group were offered one to two telephone sessions. Participants in the high-risk group were offered three to four sessions. The first session was held as soon as possible after inclusion. The duration of the follow-up period was flexible but ended when the participant reached 6 months of consecutive sick leave or had RTW in his/her contracted work hours for four consecutive weeks.

#### Training and fidelity evaluation

The MI training was a 6-day course provided by a clinical psychologist (RH) and psychiatrist (GB). The caseworkers were offered group mentoring from another psychologist, every other month during the intervention period. All were experienced MI trainers. In addition, the caseworkers could request individual feedback based on submitted recordings of MI sessions. The eight main caseworkers providing the MI were all women, aged between 27 and 65 years, with 2–20 years of work experience. The SVAI training was a 5-day course provided by a consultant physiotherapist and work and health researcher (GS). The physiotherapists were offered online group mentoring approximately every month during the intervention period. The four main physiotherapists providing the SVAI were all women, aged between 28 and 45 years, with 4–21 years of work experience.

To assess the fidelity of the MI and SVAI, we recorded intervention sessions of approximately 10% of the participants receiving the interventions. In addition, the physiotherapists documented the follow-up they provided for each participant in an intervention log. The recordings of the MI sessions were scored by an independent MI analysis centre using the Motivational Interviewing Treatment Integrity code.^24^

### Data collection

We obtained data from national registries including information on: sick leave benefits, sick leave certificates, disability pensions and contracted work hours. The primary outcome was the number of sickness absence days over 6 months defined as lost workdays. In Norway, people may combine part-time disability pensions with work. Therefore, any increase in disability pensions from baseline was also counted as sick leave. To convert time on sick leave to actual time away from work we accounted for the participants’ contracted work hours and the amount of sick leave. This was summed up and converted to lost workdays, according to a 5-day working week when working full-time.

The participants completed a questionnaire at baseline covering: age, gender, education level, marital status, first language, height, weight, smoking, follow-up from employer (yes/no), conflict with employer (yes/no), work ability (single question from the Work Ability Index, 0–10 scale),^25^ work satisfaction (single question from the original version of the ÖMPSQ, 0–10 scale^26^), physical activity in the previous week (single question from the Musculoskeletal Health Questionnaire (MSK-HQ), 0–7 scale^27 28^), musculoskeletal health (MSK-HQ, 0–56 scale^27 28^), health literacy (Health Literacy Scale Questionnaire 12, 12–72 scale^29^) and self-rated health (EuroQol Visual Analogue Scale 0–100), in addition to the Keele STarT MSK tool,^22 23^ and the ÖMPSQ-SF.^21^ For all scale variables, low values indicate low levels of the construct. To assess the representativeness of the trial sample, we obtained anonymised registry data covering sex, age, occupation, and contracted work hours from all eligible candidates.

### Sample size

The sample size calculation was conducted for the number of sickness absence days over 6 months. There is no agreed minimal important difference for this outcome described in the literature. Therefore, we based the power calculations on results from trials evaluating similar interventions for people with musculoskeletal disorders (the UK SWAP trial,^13^ and a trial conducted in Sweden with a similar welfare system to Norway^30^). Based on these trials we anticipated a difference of 10 days (two full work weeks) over 6 months between UC and UC+MI or UC+SVAI, with an expected SD of 28 days. Given a statistical power of 80% and a two-tailed 5% significance level, we estimated needing 125 participants in each arm. After adjustment for expected skewed data and 5% loss to follow-up we estimated needing to include 150 participants in each trial arm.

### Data analyses

Analyses were performed in accordance with the published statistical analysis plan,^15^ in Stata/MP V.16.1 by the first and last author (FA and BEØ) and a statistician (MCS) masked to treatment allocation. We performed descriptive statistics on all data and investigated the distributions of the variables with histograms and the Shapiro-Wilk and skewness-kurtosis tests for normality.

#### Analyses of differences in the primary outcome

The primary intention-to-treat (ITT) analysis was conducted using robust multiple linear regression, with sickness absence days as the dependent variable. We entered the ‘trial-arms’ and possible confounders (predefined in the statistical analysis plan^15^) as independent variables. To include participants with missing values, 10 data sets were imputed using multiple imputations by chained equations, following the guidance by White and colleagues.^31^ Auxiliary variables included in the imputation model were: duration of sick leave at baseline, Keele STarT MSK risk group, ÖMPSQ-SF risk group, work satisfaction and self-rated health. We checked normal probability plots, residual scatterplots and values for leverage, Cook’s distance and variance inflation factors to see if the assumptions for linear regression were met. If necessary, variables were log-transformed.

In addition, we conducted a complete case analysis. Unadjusted analyses of the differences in median and mean sickness absence days were investigated with Mann-Whitney Wilcoxon tests and t-tests. We conducted 10 000 bootstrap samples to estimate 95% CIs for the median value of sickness absence days in each trial arm.

All the statistical tests were two-sided and a p value<0.05 was regarded as statistically significant. We did not adjust for multiple comparisons as the trial evaluated the difference between UC+MI and UC+SVAI versus UC separately,^18^ and a single model was used for the multiple analyses.

#### Sensitivity analyses

Three unadjusted sensitivity analyses were performed: (1) excluding the participants recruited during the internal pilot, (2) excluding participants who had RTW for >50% of their contracted work hours 1 week after baseline (as the protocol stated that the MI and SVAI should not be delivered to participants who had RTW for >50% before the first session), (3) a moderation analysis to test if the COVID-19 pandemic moderated the effectiveness of MI or SVAI. The analysis was conducted using robust multiple linear regression including ‘trial arms’, and a variable indicating if the 6-month follow-up was completed before or after the government-imposed restrictions due to the COVID-19 pandemic, plus interaction terms between these two variables.

### Patient involvement

Patient representatives with various musculoskeletal disorders were involved in the planning of the trial. They provided guidance related to the relevance, aim and conduct of the trial and helped with the wording of the information provided to trial participants.

## Results

### Enrolment

**Figure 1:**
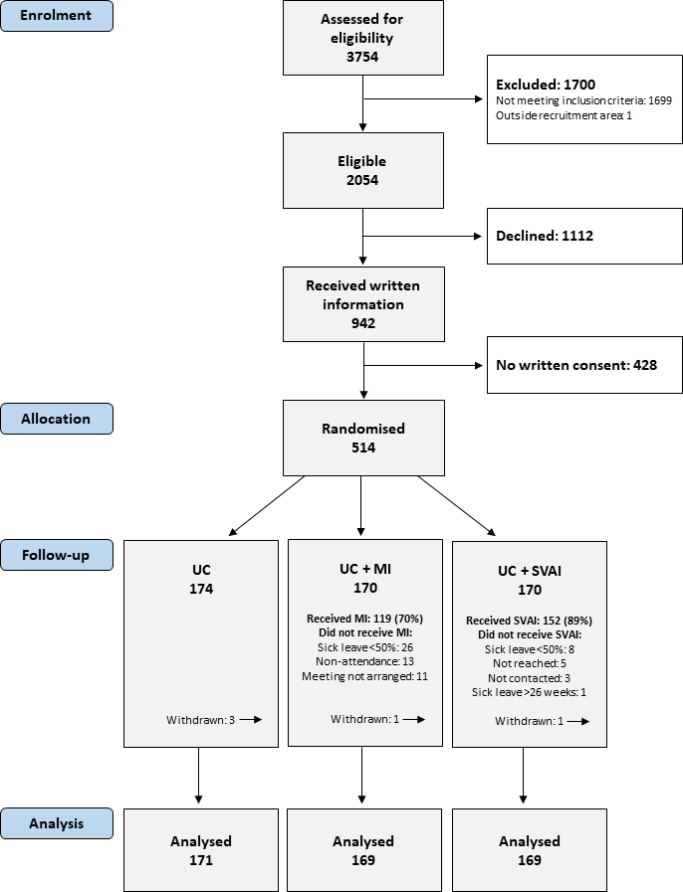
Flow chart of inclusion and follow-up of trial participants. MI, motivational interviewing; SVAI, stratified vocational advice intervention; UC, usual case management.

A total of 514 workers participated in the trial. An overview of enrolment and flow of participants is shown in online supplemental appendix 2 and figure 1. No major changes were made during the pilot phase, and the pilot participants (n=101) were included in the analyses. Recruitment was halted between 12 March 2020 and 30 March 2020 due to COVID-19 containment strategies, and we made some minor trial modifications (listed in online supplemental appendix 3). Five participants withdrew from the trial. Due to the GDPR, we could not obtain registry data from withdrawals, leaving 509 (99%) participants for the ITT analyses. No adverse events were reported during the trial.

### Baseline characteristics of the participants

Baseline characteristics are summarised in table 1. The median age of participants was 49 years (range 24–66 years) and 57% were women. Totally, 341 participants (66%) worked in full-time positions, and 315 (62%) were on full sick leave at baseline. Overall, the baseline characteristics were similar across the three trial arms. The trial sample was representative regarding age, sex and occupation compared with all eligible candidates (online supplemental appendix 4).

**Table 1:** Baseline characteristics of participants

Characteristic	Missing n (%)	UC (n = 174)	UC+MI (n = 170)	UC+SVAI (n = 170)
Age (years), median (IQR)		49 (40–55)	49 (41–56)	49 (41–56)
Women, n (%)		94 (54)	99 (58)	100 (59)
Married/living with partner, n (%)	1 (0.2)	120 (69)	119 (70)	119 (70)
Norwegian as first language, n (%)	2 (0.4)	151 (87)	154 (91)	145 (86)
Education, n (%)				
Compulsory education		21 (12)	14 (8)	20 (12)
High school		92 (53)	95 (56)	84 (49)
College or university <4 years		40 (23)	46 (27)	49 (29)
College or university ≥4 years		21 (12)	15 (9)	17 (10)
Health literacy* (12–72), median (IQR)	49 (10)	51 (44–60)	53 (45–59)	52 (44–59)
Smokers, n (%)		39 (22)	35 (21)	36 (21)
Body mass index (kg/m^2^), median (IQR)	13 (3)	28 (24–31)	27 (24–31)	27 (24–31)
Days of physical activity previous week, n (%)	1 (0.2)			
0 days		65 (37)	54 (32)	64 (38)
1-2 days		46 (26)	43 (25)	39 (23)
3-4 days		38 (22)	45 (27)	41 (24)
5-7 days		25 (14)	27 (16)	26 (15)
Musculoskeletal health† (0–56), mean (SD)	21 (4)	27 (9)	27 (8)	27 (8)
Work ability‡ (0–10), median (IQR)	3 (0.6)	2 (0–5)	3 (1–5)	3 (0–5)
ÖMPSQ-SF§ (≥60), n (%)		65 (37)	55 (32)	59 (35)
Keele STarT MSK tool (0–12)				
High risk (≥9), n (%)		61 (35)	49 (29)	48 (28)
Medium risk (5–8), n (%)		85 (49)	86 (51)	98 (58)
Low risk (<5), n (%)		28 (16)	35 (21)	24 (14)
High-risk for long-term sick leave¶, n (%)		38 (22)	36 (21)	35 (21)
Work satisfaction** (0–10), median (IQR)	1 (0.2)	8 (6–9)	8 (7–9)	8 (6–9)
In conflict with employer, yes n (%)	4 (0.8)	6 (3.5)	5 (3.0)	14 (8.3)
Followed-up by employer, n (%)	7 (1)			
No follow-up		65 (38)	72 (44)	72 (43)
Dialogue meeting or follow-up plan		64 (37)	53 (32)	65 (38)
Dialogue meeting and follow-up plan		44 (25)	40 (24)	32 (19)
White-collar workers, n (%)		58 (33)	56 (33)	61 (36)
Blue-collar workers, n (%)		116 (67)	114 (67)	109 (64)
Work, n (%)				
Full-time		120 (69)	110 (65)	111 (65)
Part-time 50–99% of full work hours per week		39 (22)	53 (31)	48 (28)
Part-time <50% of full work hours per week		15 (9)	7 (4)	11 (6)
Graded disability pension††, yes n (%)	5 (1)	15 (9)	12 (7)	9 (5)
Sickness absence days previous year (work days‡‡), median (IQR)	5 (1)	38 (30–50)	35 (31–50)	36 (26–50)
Duration of consecutive sick leave at baseline (calendar days), median (IQR)	5 (1)	51 (50–55)	51 (50–55)	51 (49–56)
Sick leave at baseline, n (%)	5 (1)			
Full-time sick leave		103 (60)	109 (65)	103 (61)
Sick leave 50–99% of contracted work hours		65 (38)	54 (32)	63 (37)
Sick leave <50% of contracted work hours		3 (2)	6 (4)	3 (2)
Area of body pain, n (%)	14 (3)			
Lower limb		6 (4)	18 (11)	15 (9)
Upper limb		30 (18)	30 (18)	30 (18)
Neck		12 (7)	12 (7)	10 (6)
Back		34 (20)	42 (25)	43 (26)
Multisite pain		12 (7)	8 (5)	10 (6)
Joint disorders		20 (12)	13 (8)	10 (6)
Fractures		14 (8)	16 (10)	11 (7)
Other		40 (24)	26 (16)	38 (23)

The distribution was skewed for all continuous variables, except for the MSK-HQ.

*Measured with the Health Literacy Scale Questionnaire.

†Measured with the Musculoskeletal Health Questionnaire (MSK-HQ).

‡Measured with a single question from the Work Ability Index.

§ÖMPSQ-SF: The Örebro MSK Pain Screening Questionnaire Short Form (0–100).

¶High-risk group in the MI-NAV trial: ≥60 on the ÖMPSQ-SF and ≥9 on the Keele STarT MSK Tool.

**Work satisfaction: 0=not satisfied at all, 10=totally satisfied.

††Individuals who work part-time and receive a graded disability pension.

‡‡Lost workdays due to sick leave, adjusted for work hours per week and amount of sick leave.

MI, motivational interviewing; n, number of participants; SVAI, stratified vocational advice intervention; UC, usual case management.

### Intervention delivery

The number of sessions and duration of the MI and SVAI interventions are listed in table 2. Following the COVID-19 pandemic 22 (10%) of the MI sessions were provided by telephone or video call. All the SVAI sessions were provided by telephone and none of the physiotherapists attended workplace meetings.

**Table 2:** Summary of delivery of MI and SVAI

	UC+MI (n=170)	UC+SVAI (n=170)
Received intervention, n (%)	119 (70)	152 (89)
Number of sessions*, n (%)		
One session	3 (2)	13 (8)
Two sessions	106 (62)	106 (62)
Three sessions	n.a.	10 (6)
Four sessions	n.a.	19 (11)
Days until first session*, mean (SD)	21 (13)	6 (5)
Intervention period* (days), mean (SD)	36 (17)	50 (27)
Intervention period low/medium-risk group	n.a.	42 (21)
Intervention period high-risk group	n.a.	74 (30)
Duration of first session† (min), median (IQR)	41 (26–45)	45 (35–60)
Duration of follow-up sessions‡ (min), median (IQR)	46 (45–49)	25 (20–30)

*We did not have data on 4 of the participants receiving SVAI and 10 participants receiving MI.

†We only had data from 15 MI sessions.

‡We only had data from 6 MI sessions.

%, per cent of participants randomised to the intervention arm; MI, motivational interviewing; n, number; n.a., not applicable; SVAI, stratified vocational advice intervention; UC, usual case management.

### Primary outcome

**Figure 2:**
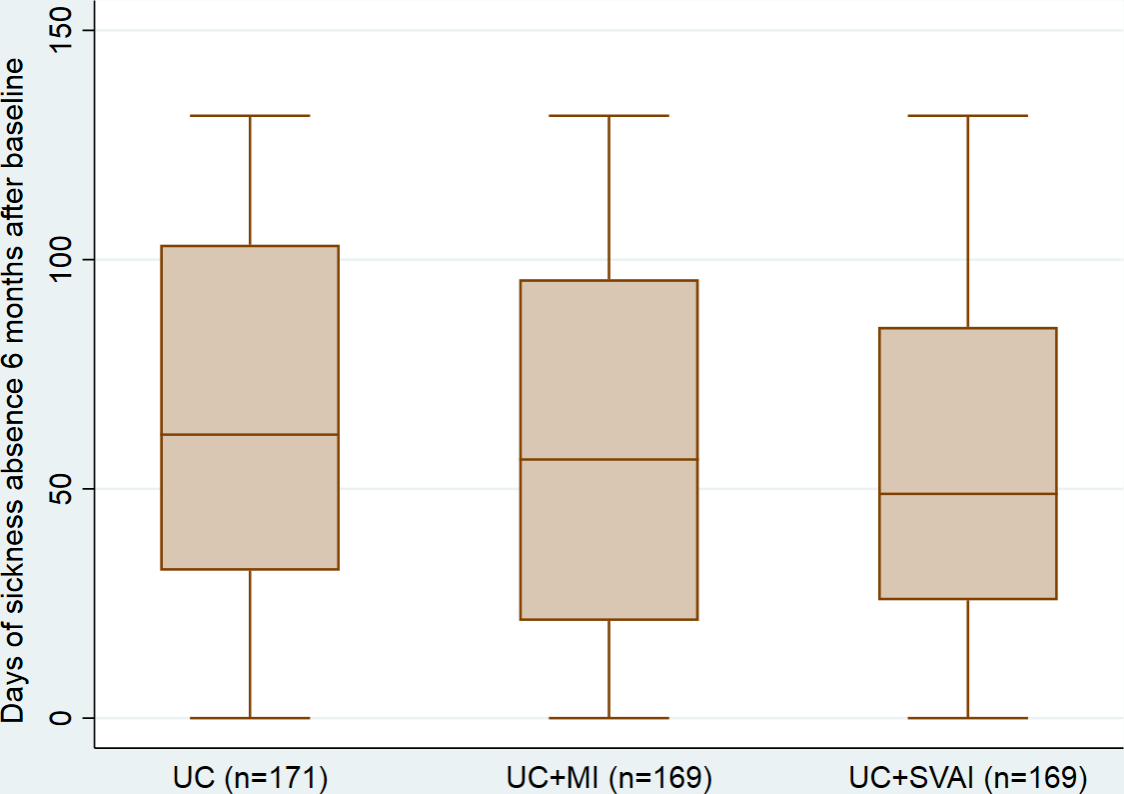
Distribution of sickness absence days (median, IQR and range) for participants in each of the trial arms. MI, motivational interviewing; SVAI, stratified vocational advice intervention; UC, usual case management.

Three participants did not have any sickness absence from baseline to 6 months follow-up (some participants were late in answering the baseline questionnaire and had RTW before inclusion in the trial) (figure 2). Thirteen participants reached the maximum amount of sickness absence possible during the follow-up period (131 days). The distribution of sickness absence days from baseline to 6 months follow-up was skewed in all three trial arms.

#### Unadjusted analyses

The UC+MI arm had 6 fewer median days of sick leave compared with the UC arm (not statistically significant (ns)) and the mean difference was 7 fewer days (95% CI −16 to 2) (ns) (table 3). The UC+SVAI arm had 13 fewer median days of sick leave compared with the UC arm (p=0.04), the mean difference was 9 fewer days (95% CI −17 to −0.1) (p=0.04) compared with UC (table 3).

**Table 3:** Unadjusted analyses. Sickness absence days over 6 months, comparison between UC and UC+MI or UC+SVAI

	UC			UC+MI			UC+SVAI		
	n	Mean (SD)	Median (95% CI)	n	Mean (SD)	Median (95% CI)	n	Mean (SD)	Median (95% CI)
ITT	171	66 (41)	62 (52–71)	169	59 (41)	56 (43–70)	169	57* (38)	49* (38–60)
Low/medium-risk group	135	63 (41)	58 (48–69)	133	55 (41)	45 (29–61)	134	55 (37)	48 (37–59)
High-risk group	36	76 (40)	79 (60–97)	36	73 (42)	71 (52–90)	35	66 (40)	61 (33–90)
Sensitivity analysis 1	137	66 (41)	62 (49–74)	139	58 (41)	57 (43–71)	132	58 (39)	53 (41–65)
Sensitivity analysis 2	163	68 (40)	65 (57–74)	158	62 (41)	59 (47–71)	154	59 (37)	54 (43–65)

ITT, intention-to-treat analysis (five missing: three in UC arm, one in UC+MI arm, one in UC+SVAI arm).

Sensitivity analysis 1: excluding pilot participants.

Sensitivity analysis 2: excluding participants who returned to work ≥50% within 1 week after baseline.

*Statistically significant difference (p<0.05) compared with UC only, tested with t-test or Mann-Whitney Wilcoxon test.

95% CI, 95% confidence interval (estimated with 10 000 bootstrap resamples); MI, motivational interviewing; n, number of participants in analysis; SVAI, stratified vocational advice intervention; UC, usual case management.

#### Adjusted analyses

The assumptions for linear regression were met apart from several outliers. We conducted robust linear regressions to reduce the outliers’ effect on the estimates (table 4). The primary imputed analysis (n=509) showed that the UC+MI arm had 7 fewer days of sickness absence (95% CI −15 to 2) compared with UC (ns). The UC+SVAI arm also had 7 fewer days (95% CI −16 to 1) compared with the UC arm (ns). In the complete case analysis (n=479) the difference was 9 fewer days for both the UC+MI arm (95% CI −18 to −0.4) and the UC+SVAI arm (95% CI −18 to −0.7), compared with the UC arm (p<0.05).

**Table 4:** Robust linear regression analyses. Estimation of differences in sickness absence days over 6 months between UC and UC+MI or UC+SVAI

Variable	Unadjusted ITT analysis (n=509)			Adjusted complete case analysis* (n=479)			Adjusted primary ITT analysis with imputations*† (n=509)		
	Coef. B	95% CI		Coef. B	95% CI		Coef. B	95% CI	
UC+MI	−7.3	−16.6	1.9	−**9.2‡**	−**17.9**	−**0.4**	−6.6	−15.0	1.8
UC+SVAI	−**9.3‡**	−**18.5**	−**0.1**	−**9.4‡**	−**18.0**	−**0.7**	−7.0	−15.4	1.4
Sex, male				11.2‡	3.8	18.7	11.8‡	4.6	19.1
Age				−0.1	−0.4	0.3	−0.0	−0.4	0.3
Secondary school§				2.6	−9.6	14.8	1.9	−9.7	13.5
Higher education <4 years§				2.8	−10.3	16.0	2.9	−9.7	15.5
Higher education ≥4 years§				−11.0	−26.9	4.9	−10.3	−25.4	4.8
Meeting or follow-up plan¶				−7.2	−15.3	0.9	−5.6	−13.5	2.2
Meeting and follow-up plan¶				−5.0	−14.4	4.4	−3.5	−12.6	5.7
Physical activity 1–2 days**				0.7	−8.8	10.1	1.1	−8.1	10.3
Physical activity 3–4 days**				6.5	−3.2	16.3	3.5	−4.1	18.0
Physical activity 5–7 days**				8.1	−3.3	19.5	7.0	−4.1	18.0
Work ability††				−3.5‡	−5.0	−2.1	−3.8‡	−5.2	−2.4
Musculoskeletal health‡‡				−0.8‡	−1.3	−0.3	−0.7‡	−1.2	−0.02
Sickness absence days previous year§§				19.5‡	13.1	25.8	19.1‡	12.9	25.3

n, number of participants in analysis (ITT analysis: UC n=171, UC+MI n=169, UC+SVAI n=169, complete case analysis: UC n=158, UC+MI n=157, UC+SVAI n=159)

*Multiple robust linear regression analyses adjusted for predefined possible confounding factors.

†Values for missing on the independent variables were imputed with multiple imputations by chained equations with 10 imputations. Imputations were not conducted for the five missing outcome values.

‡p<0.05.

§Education: dummy variables compared with compulsory education.

¶Follow-up from employer, dummy variables compared with no follow-up.

**Physical activity 1 week prior to baseline, dummy variables compared with no physical activity.

††Measured with single question from the Work Ability Index (0–10).

‡‡Measured with the Musculoskeletal Health Questionnaire (0–56).

§§Number of days away from work due to sickness absence 12 months prior to baseline, logarithmic transformed variable.

Coef., Coefficient.; ITT, intention-to-treat; MI, motivational interviewing; SVAI, stratified vocational advice intervention; UC, usual case management.

#### Sensitivity analyses

We only observed minor differences in the sensitivity analyses compared with the ITT analysis (table 3). The interaction terms in the moderation analysis to test if the COVID-19 moderated the effect of MI or SVAI had large CIs and were not statistically significant.

## Discussion

### Principal findings

The MI-NAV trial showed a 7-day reduction in sickness absence over 6 months of adding either MI or SVAI to UC, for workers on sick leave due to musculoskeletal disorders. However, the results were estimated with low precision reflected in wide CIs, the differences were smaller than anticipated and not statistically significant.

### The MI intervention compared with previous studies

Although our findings were not statistically significant, they are in line with findings from a Canadian cluster RCT, indicating that MI could reduce sickness absence among people with musculoskeletal disorders.^32 33^ In the Canadian trial MI was added to interdisciplinary rehabilitation at a rehabilitation centre, and reduced the recurrence of wage replacement benefits by 5% over 12 months for employed workers.^32^ In the Canadian study MI was provided by occupational and exercise therapists.^32 33^ However, the role of a NAV caseworker differs from a healthcare professional and they do not have medical training. A recent study, interviewing workers on sick leave who had received MI from NAV caseworkers, showed that although the workers had negative expectations to the NAV (because of their role as gatekeepers to sickness benefits), they developed a good relationship to the NAV caseworkers and experienced the MI sessions as positive and helpful in the RTW process.^34^ Similar findings have been shown among workers on sick leave in Sweden,^35^ and an RCT from the USA has shown that MI training can improve working alliance between clients and RTW counsellors.^36^

The NAV caseworkers in our trial provided the MI in addition to their usual workload. This may explain the long duration from baseline until the first MI session, and was the main reason that 30% of the participants in the MI arm did not receive MI. Four caseworkers dropped out during our trial due to an otherwise high workload or lack of MI experience.^17^ The evaluation of the 21 recorded MI sessions from the MI-NAV trial revealed that although the NAV caseworkers had high adherence to the MI guideline, they had low MI proficiency levels throughout the trial.^17^ This is in line with findings from a similar Norwegian study.^37^ These factors may have reduced the effectiveness of the MI intervention in our study.

### The SVAI compared with previous studies

The results from the MI-NAV trial support the findings of the SWAP trial indicating that vocational advice could reduce sickness absence among workers with musculoskeletal disorder. However, our results were not statistically significant after adjusting for possible confounders. The SWAP trial showed a reduction of 5 days of sickness absence over 4 months of adding a vocational advice intervention to best current primary care in the UC.^13^ In both trials the vocational intervention was provided by physiotherapists mostly by telephone, and a median of two sessions was provided. However, the SVAI was delivered as stratified care with one to two sessions provided for the low/medium-risk group, and three to four sessions for the high-risk group. The SWAP intervention, on the other hand, was delivered as stepped care, with the possibility of providing more sessions if necessary. In the SWAP trial 57% of the participants were doing their usual job, while the participants in the MI-NAV trial had been on sick leave for more than seven consecutive weeks. Therefore, the participants in our trial might have needed more RTW support, compared with the workers in the SWAP trial and it might have been preferable to deliver the intervention as stepped care (with the possibility of providing more sessions to participants who needed more help to RTW).

Although the SVAI was mainly delivered according to protocol, some intervention elements were poorly implemented.^16^ The physiotherapists did not attend workplace meetings or arrange face-to-face meetings with participants. They also had few contacts with important RTW stakeholder such as NAV caseworkers, employers and general practitioners.^16^ Previous studies have shown that cooperation between RTW stakeholders is important,^38^ and the physiotherapists limited liaison with stakeholders may have reduced the effectiveness of the SVAI in our trial.^16^

### Strengths and limitations of the MI-NAV trial

The multi-arm RCT design made it possible to compare two additional interventions with a single UC arm, optimising the use of limited research resources.^39^ We obtained detailed national registry data for 99% of the trial participants and conducted thorough fidelity evaluations. To reduce the risk of intervention contamination, the NAV offices had not trained their caseworkers in MI prior to the trial. The caseworkers were instructed not to use MI in usual follow-up of people on sick leave with musculoskeletal disorders. The physiotherapists delivering the SVAI only provided vocational follow-up to participants randomised to the SVAI arm.

Our trial had limitations in addition to those previously discussed. First, we had a low inclusion rate of 25% of those eligible. However, registry data showed that our sample was representative of the larger population regarding important factors associated with sick leave (sex, age and occupation). Furthermore, there is no agreed minimal important difference for sickness absence. A 7-day difference may be considered an important effect. However, our trial was not powered to detect this difference as statistically significant. Large variability in the data may also have reduced the statistical power of our trial. Another limitation is that the trial was not powered to perform subgroup analyses to detect possible differences in effects of adding MI or SVAI to UC for the low/medium-risk group and the high-risk group separately, or to compare UC+MI with UC+SVAI. This would have required an unrealistically large sample size. The participants in the UC+MI arm and the UC+SVAI arm received more follow-up compared with participants in the UC arm. Therefore, we cannot rule out that it was the extra follow-up and not the intervention elements that facilitated RTW. This will be controlled for in a recent RCT using the same MI intervention as the MI-NAV trial.^40^ Lastly, possible intervention contamination from the NAV caseworkers was not evaluated in the process evaluation of the trial. However, the risk for contamination with the UC arm was low since NAV caseworkers usually do not convene a meeting with workers during the first 6 months of sick leave.

## Conclusion

Adding MI or SVAI to UC for workers on sick leave for at least 7 weeks due to musculoskeletal disorders, reduced sickness absence by an average of 7 workdays over 6 months. The differences were not statistically significant, and the results were uncertain due to wide CIs. Efforts should be made to improve implementation of the MI and SVAI in future trials, and it might be preferable to provide the interventions as stepped care. The acceptability of the MI and SVAI to those providing and receiving the interventions should be investigated.

10.1136/oemed-2022-108637.supp2 Supplementary data

## Data Availability

Data are available upon reasonable request. Requests to access data should be addressed to the last author: brielo@oslomet.no. Anonymised individual participant data (including data dictionary) will be available on request, from January 2023 to December 2028, to researchers who provide a methodologically sound scientific proposal that has been approved by an ethics committee and by the scientific board of the MI-NAV study.
