# Supplementary material for: Effectiveness of adding motivational interviewing or a stratified vocational advice intervention to usual case management on return to work for people with musculoskeletal disorders: the MI-NAV randomised controlled trial
Source: Occup Environ Med. 2022 Nov 25;80(1):42–50. doi: 10.1136/oemed-2022-108637 (PMC9763188; doi:10.1136/oemed-2022-108637)
Supplement: Supplementary data [file oemed-2022-108637supp001.pdf]

APPENDIX 1: INTERVENTIONS IN THE MI-NAV TRIAL

Usual case management (UC) according to guidelines from the Norwegian Labour and Welfare Administration (NAV)

All Norwegian citizens and people working in Norway are entitled to health care through the Norwegian National Insurance Scheme. Workers on sick leave are entitled to full wage replacement benefits for up to 12 months. The first 16 days are covered by the employer, the rest by the social security system administered through the NAV. To be entitled to sickness benefits from the NAV a sick note is required, usually issued by a medical doctor. Employers and employees are obliged to cooperate to try to prevent long-term sickness absence. During the first six months of sick leave the employer has the main responsibility for follow-up and should make a follow-up plan in cooperation with the worker within the first four weeks of sick leave. The plan should include information about the employee's work duties, workability, and possible work adaptations. Within week eight of sick leave, the employee should start work-related activity (unless it is not possible due to medical reasons). If the worker is still on full-time sick leave after eight weeks, the NAV may request documentation that work related activity is not possible. The employer is responsible for arranging a dialogue meeting with the employee within week seven of fulltime sick leave (unless it is clearly unnecessary). The purpose of the meeting is to prevent long-term sickness absence and discuss if workplace modifications are required. Within six months of sick leave the local NAV office is responsible for arranging a second dialogue meeting, including the employee, employer, and sick-leave certifier (if appropriate). The second dialogue meeting can be arranged earlier if requested by any of the parties.

Motivational interviewing (MI) according to the protocol

The MI intervention was a replication of an intervention, evaluated in a Norwegian trial conducted concurrently in Trondheim (Aasdahl et al. 2018). MI is a practical tool for counselors developed by William Miller and Stephen Rollnick to help people change. It is rooted in the person-centered approach of Carl Rogers and is inspired by several social and behavioral models. MI is based on the principle that people have the resources within themselves to change (self-determination theory). Motivation for change is activated through the person`s own change talk.

The participants randomised to the UC+MI arm were offered two face-to-face sessions of MI from NAV caseworkers, in addition to UC. The sessions could last up to one hour. The first session was delivered at a local NAV office as soon as possible after enrolment in the study, and the second session was held 2 weeks later. The NAV caseworkers followed a MI guideline developed for return to work (RTW) by Gunnhild Bagøien (a psychiatrist and member of the motivational interviewing network of trainers) and Roger Hagen (a clinical psychologist). The guideline was based on MI principles to build a collaborative relationship with the participants, including communication skills such as asking open-ended questions, providing reflections, and summaries to evoke and enhance change talk. In the first session, an agenda was set in cooperation with the participant through 'agenda mapping' (the participant decided the agenda for the conversation from a menu of topics, based on what they considered to be most relevant for their situation). The participants' readiness to return to work was assessed (using the MI tools: 'importance ruler' and 'confidence ruler'), and the intervention was tailored according to motivational stage (stages of change). If the participant

Aanesen F, *et al. Occup Environ Med* 2022; 80:42–50. doi: 10.1136/oemed-2022-108637

was ambivalent about RTW, the pros and cons of sickness absence were explored in an accepting and compassionate manner. In the second session, the participant's current work situation, obstacles to RTW and previous attempts at RTW were discussed. The caseworkers provided information about available RTW support from the NAV in a MI-consistent manner. If the participant was ready for RTW, the NAV caseworker offered to help them develop an action plan for RTW. Summaries of each session were made available to the participants on the NAV's secure online communication platform.

Stratified vocational advice intervention (SVAI) according to the protocol

Stratification

We used the 10-item version of the Örebro Musculoskeletal Pain Screening Questionnaire Short Form (ÖMPSQ-SF) (Linton et al 2011), and the 10-item Keele STarT MSK Tool (Dunn et al. 2021), to stratify the participants into two risk groups of long-term sick leave. Participants with ≥9 on the Keele STarT MSK Tool and ≥60 on the ÖMPSQ-SF were stratified to a 'high-risk group', all others were stratified to a 'medium/low-risk group'. The cut-off points were based on preliminary data from a prospective cohort study conducted as part of the MI-NAV project. The preliminary results from this cohort study showed that the combination of these tools had the greatest accuracy in distinguishing between short versus long-term sick leave in workers on sick leave due to musculoskeletal disorders in Norway (unpublished results). The ÖMPSQ-SF assesses five psychosocial risk factors related to future disability: 1) self-perceived function, 2) pain experience, 3) distress, 4) fear-avoidance beliefs, and 5) RTW expectancy. Sum scores range from 1-100 points with higher scores indicating higher estimated risk of future work disability. The Keele STarT MSK tool consists of 10 items assessing: pain intensity, pain self-efficacy, pain bothersomeness, disability, comorbid pain, expected duration of the condition, self-perceived health, depression, fear avoidance and pain duration (during the last two weeks). Sum scores range from 0-12 points, with values from 0-4 points indicating low risk, 5-8 points indicating medium risk, and 9-12 points indicating high risk for poor prognosis.

Intervention

The SVAI was a modified version of an intervention developed for the SWAP trial (Sowden et al. 2019). The theoretical underpinning of the intervention was social cognitive theory, self-determination theory and the common-sense model of self-regulation (Aanesen et al. 2021).

The participants stratified to the low/medium-risk group were offered up to two telephone sessions. The sessions could last up to one hour. Participants in the high-risk group were offered three to four sessions, the first by telephone, the remaining sessions either by telephone or face-to-face, including an optional workplace meeting. The first session was held as soon as possible after the baseline assessment. The duration of the follow-up period was flexible but ended when the participants reached 6 months of consecutive sick leave or had RTW in their contracted work hours for 4 consecutive weeks. During the first session, the physiotherapists followed a semi-structured conversation guide with open-ended questions to clarify the participants' work and health situation and identify obstacles to RTW. During the sessions, the physiotherapists provided evidence-based advice on the management of musculoskeletal disorders and supported problem-solving to overcome modifiable obstacles to RTW. The follow-up provided by the physiotherapists was tailored according to the participants' needs and individual RTW barriers. They collaborated with the participants to decide goals for RTW, developed and implemented action plans, facilitated communication,

collaboration and coordination with stakeholders and signposted to other services if necessary.

Information leaflet for participants receiving SVAI

Research study aiming to help sick listed people with musculoskeletal disorders back to work

The research study is led by researchers from the MUSK health research group at Oslo Metropolitan University, OsloMet. The aim of the study is to find ways to help people who are sick-listed with musculoskeletal disorders. The study will test two different types of dialogue based interventions. All participants will receive usual follow up from NAV in addition to the interventions given in the study. They can also receive medical treatment from other health care professions during the trial if they wish.
You can find more information about the study on the following link:
https://www.muskhealth.com/minav3

Vocational advice from physiotherapist

You have agreed to participate in the study and have been randomly assigned to receive vocational advice from a physiotherapist. The physiotherapist will discuss topics related to your health and work situation and try to help you get back to work.
The physiotherapist does not have any connection to the NAV or your employer.
Confidentiality: The physiotherapist is bound by law to follow high standards of confidentiality and can't share information with your employer or the NAV without your consent.

What can the physiotherapist do?

- Discuss concerns you have regarding health and work.
- Help you make a return to work plan.
- Identify barriers for returning to work.
- Suggest actions to help you return to work, e.g. suggest adaptions to your work situation, give advice regarding treatment, and how to cope with your health problems.

If you consent the physiotherapist can also:

- Collaborate with you doctor or other health care professionals.
- Collaborate with your employer.

What can you do to get back to work sooner?

- Keep in contact with your workplace and colleagues. You and your employer are responsible for making a follow up plan. Your employer is also responsible for making adaptions to your work if this is necessary for you to be able to return. Your duty is to collaborate with your employer to make this possible.

Appendices, The MI-NAV trial

- Talk to your doctor or physiotherapist about how they can help you get back to work. You know your work best. Discuss which parts of your job you can do with your health problem. Ask for treatment designed to get you ready for work.
- Gradually increase your activity level. Start with the activities you find easy, and do a bit more each day. You will have good days and bad days. Try to keep active also on the bad days. Vary between rest and activity. It is common to have set backs - so don't give up!

Evidence based information about work and health

- Research shows that in general work is good for mental and physical health. Work is important for self-esteem and quality of life.
- Being absent from work can have negative effects for health and wellbeing.
- Musculoskeletal disorders are very common and all of us suffer these kinds of problems at some time in our life.
- The pain can be very distressing and may make life difficult, but there is usually no serious disease or lasting damage. Most episodes end quickly, though some symptoms may continue or come back from time to time.
- We have good evidence that returning to work as soon as possible helps recovery, and is the best way to avoid long-term sickness absence.

There are many unhelpful myths about health and work, which can cause unnecessary fear and uncertainty:

1. *Common health problems are caused by work.* Usually they are not. Everyone has these kinds of problems. Some type of work can make the symptoms feel worse, but usually work does not cause the problem.
2. *Work will make my condition worse:*
   Most people with musculoskeletal disorders can continue working. In many cases going back to work can help you feel better.
3. *You should not go back to work until you are fully recovered:*
   Usually the opposite is true. Work can be part of treatment. Getting back to work and activity can help you recover. Adjustments to your work can make it possible to return to work sooner.
4. *A sick certificate means that you must not work:*
   A sick certificate is not an order from your doctor to stay away from work, it only means you are entitled to sick pay. You can return to work as soon as you are ready.

Conversation guide for the SVAI physiotherapists

INTRODUCTION

Suggested introduction in first telephone contact

My name is … I am a physiotherapist from the MI NAV research project, am I talking to….. ? Is this a good time to talk?
IF NO: Make new appointment.
IF YES: Thank you for participating in our study. Could you please tell me your address and date of birth?
Is it OK that I tell you a bit about the research project and the part of the study you have been assigned to?
The research project is led by researchers from the MUSK health research group at OsloMet (Oslo Metropolitan University). The aim of the study is to find ways to improve follow-up for people who are on sick leave with musculoskeletal disorders. In the study we are testing two different interventions and compare these to usual follow-up from the NAV. I am calling you because you are in the group who will get help from a physiotherapist trained to give vocational advice.
My job is to help you get back to work, but I do not have any connection with the NAV or with your employer.
Do you have any questions regarding this?
During our conversation today I wish to get to know your situation and any problems you have regarding returning to work.
I am bound by law to keep anything you tell me confidential and can't share information with NAV or your employer without your consent.
I know that you have answered a questionnaire for this project, but I don't have access to your answers.
Is it OK if I ask you some questions regarding your health and your situation at home and at work?

CLARIFY CURRENT WORK AND HEALTH SITUATION

Ask open questions and use reflection to build rapport and clarify the participants health and work situation.
Gather information regarding all the questions in red on this form, the rest of the questions can be asked when appropriate/if you need more information.
Write notes for every topic. You do not need to follow the order of the form, but you should cover all the topics during the conversation

WORK SITUATION

**Sick-listed date**	**% sick listed**	**End of sick certificate**

Can you describe your current work situation?

- *What is your current occupation/title?*
- *What are your contracted hours of work? (contract work %, days and hours at work, shift pattern)*
- *Do you usually work more than your contracted hours?*
- *Do you usually work overtime?*
- *What does a typical day at work for you look like?/ What does your job involve? (Physical job demands, emotional and cognitive job demands).*

IDENTIFY OBSTACLES TO RETURN TO WORK

Use open ended questions, reflection and summarising to identify obstacles to return to work.
Note any identified obstacles in the action plan.

How are your symptoms /condition affecting your ability to work?

- *What do you think about working with your present pain/ symptoms?*

Appendices, The MI-NAV trial

- *Are you worried about any repeat episodes of symptoms/problems once you return to work?*
- *How do you feel about the prospect of returning to work at some point?*

What are your main concerns about RTW?

- *What would make RTW difficult now?*
- *Are there stressful elements to your job that might be difficult when you first return to work?*
- *Aside from your symptoms are there any other reasons why it would be difficult to RTW now?*

Have you had a dialogue meeting with your employer?

- Meeting held
- Meeting planned

What other contact have you had with work since you have been off sick?

What contact have you had with NAV?

Has your employer made a return-to-work plan?
Has the plan been sent to the person who gave you the sick note?
How is the plan working?

- *What are they doing at work to help?*
- *Have you discussed with your employers when you might return to work or start working more?*
- *When do you think you will go back to work/start working more hours?*
- *Do you have an occupational health service at work?*
- *Have you had involvement with them?*

How happy are you with your work and workplace?

- *How would you describe your relationship with your colleagues and employer?*
- *Did you have any conflicts with your employer or co-workers before you were sick listed?*
- *What kind of response do you expect from co-workers and supervisors when you return?*
- *Is your job at risk?*
- *Do you enjoy your job?*
- *What is it that you value about working or your job?*
- *What else does work do for you/ do you get from work?*
- *Why did you choose the job/career you did?*
- *How important to you is it to get back to work?*

OVERCOMING OCCUPATIONAL OBSTACLES TO RTW

Collaborate with the worker to problem solve and overcome obstacles (see separate obstacles and actions sheet). Note any actions in the action plan.

What could be done at the workplace to help you return to work/increase your work hours?

- *What elements / hours of your job are you already doing?*
- *What elements / hours do you think you could manage now?*
- *Are you doing lighter or modified hours or duties?*
- *Do you expect your work could be modified temporarily so you could return to work sooner? Are you able to build back into work gradually? Are light duties an option?*
- *How many hours do you think you could manage to begin with?*
- *When do you think you could start?*

Short term work goal

Long term work goal

Appendices, The MI-NAV trial

HEALTH SITUATION

Could you please tell me briefly about the main health problem that you are struggling with at the moment?

- *When did this episode of pain start?*
- *Have you had any previous episodes?*
- *Do you think there is a high risk of your current pain becoming persistent?*
- *Do you have any other important health problems?*
- *What do you think has caused your health problem?*
- *Is your pain related to an injury? Was it an injury/accident at work? Is there a litigation case or insurance claim related to the accident?*

How is your health condition affecting you day to day?

- *Are you avoiding doing anything or particularly struggling with anything?*
- *How are you sleeping?*
- *How well do you feel you are managing at the moment?*
- *What do you think would help you better manage your symptoms/ condition?*
- *How is the situation affecting your mood?*

Can you describe any treatment you are receiving or have received for your condition?

- *Ongoing treatment.*
- *Previous treatment.*
- *Are you waiting for any appointments, tests or treatments?*
- *Do you think you should be having any tests or treatment for your symptoms/condition?*
- *Do you feel you understand your condition and any treatment you are receiving?*
- *What contact have you had with the person who gave you your sick note since you were sick listed?*
- *Is anyone else helping you with your health problem?*

FAMILY SITUATION

- *Do you live alone or with somebody?*
- *Do you have any children?*
- *How old are they?*
- *Is there anything going on at home or to do with your current circumstances that would make it difficult to RTW now?*
- *What arrangements might need to be made at home in order to help you return to work? (carer, childcare, transport etc?)*

Appendices, The MI-NAV trial

Action sheet for the SVAI physiotherapists

OBSTACLES AND ACTIONS

Identify potential obstacles and actions during phone call and write them down in the action plan.

ASK FOR CONSENT BEFORE CONTACTING OTHER STAKEHOLDERS (HEALTHCARE PROFESSIONALS, EMPLOYERS OR NAV CASEWORKER)

POTENTIAL OBSTACLES	SUGGESTED ACTIONS FROM SVAI PHYSIOTHERAPIST
High severity of symptoms/health condition.<br>Comorbid health is a potential obstacle to RTW.<br>Delays in health care.<br>Lack of work focus to health care.	- Ask if participant is taking his/her medication as prescribed by their GP.<br>- Suggest that worker makes appointment to see GP.<br>- Contact health care providers in order to:<br>a) suggest an appointment/investigation<br>b) expedite an appointment<br>c) ensure the HCP facilitates RTW<br>d) post evidence based information to the health care provider
Current physical functioning not compatible with RTW.	- Suggest that participant sees a physiotherapist, if necessary help to set up appointment.<br>- Do values based goal setting.
Avoiding activities.<br><br>Unhelpful beliefs about health and work.	- Provide reassurance to participant regarding hurt and harm.<br>- Advises the participant about how to gradually increase activity and exercise and return to avoided activities.<br>- Send leaflet with evidence based information to participant.<br>- Provide evidence based information, advice and reassurance to address knowledge gaps, misconceptions or unhelpful beliefs verbally.
Current day/night rest and sleep pattern not compatible with working.	- Provide verbal information about sleep.<br>- Inform participant about online resource to deal with sleep disturbance.
Doesn't value work sufficiently to RTW.	- Use motivational interviewing to help the participant decide whether to RTW or not.<br>- Explore and built the value of work.<br>- Convey positive but realistic messages about their ability to work now or in the future.<br>- Encouraged participant to be pro-active in taking steps to resolve the situation.
Lack of or unsupportive contact with the workplace.<br>Other workplace issues.	- Suggest that participant makes contact with employer.<br>- Take direct contact with employers if participant needs help with this.<br>- Arrange and attend meeting between SVAI physiotherapist, worker and employers.<br>- Inform the NAV caseworker about the worksite meeting/visit if NAV caseworker is involved in the case.

Appendices, The MI-NAV trial

Lack of a RTW plan.	- Support participant to develop RTW plan with employers.<br>- Build participant self-efficacy to collaborate with employer to make RTW plan, e.g. help make a list of what they want to discuss with employer, roleplay meeting etc<br>- Liaise with participant and employer in developing RTW plan.
Poor implementation of RTW plan.	- Review RTW plan with participant.<br>- Ask participant to liaise with employers to commence already agreed RTW plan.<br>- Discuss with participant how they will work with employers to stick to plan, review plan, modify plan and seek help early, if needed.<br>- Liaise with participant and employer to implement existing plan.

APPENDIX 2: RECRUITMENT OF PARTICIPANTS TO THE MI-NAV TRIAL

**Year**	**2019** Internal pilot[#]								**2020**				Cov.¤							
**Month**	**Apr**	**May**	**June**	**July**	**Aug**	**Sept**	**Oct**	**Nov**	**Dec**	**Jan**	**Feb**	**Mar 1-11**	**Mar 12-31**	**Apr**	**May**	**June**	**July**	**Aug**	**Sept**	**Oct**
**Recruited**	11	17	17	34	31	33	65	50	15	46	34	28	4	22	14	4	7	24	37	21
**Total**	11	28	45	79	110	143	208	258	273	319	353	381	385	407	421	425	432	456	493	514

[#] Internal pilot including the first 101 participants
¤ Cov. COVID-19 containment strategies were implemented by the Norwegian government on the 12th of March 2020 and recruitment was halted between 12-30 of March

APPENDIX 3: TRIAL MODIFICATIONS DUE TO THE COVID-19 PANDEMIC IMPLEMENTED AFTER THE 12TH OF MARCH 2020, Planned by the trial team and reviewed and approved by the scientific board

Modification	Reason for modification	Consequence of modification
The MI intervention may be delivered by telephone or video call, rather than in-person only.	The Norwegian government implemented wide-reaching COVID-19 containment strategies on the 12th of March 2020 to decrease physical contact between individuals.	22 (10%) MI sessions were delivered by telephone or video call, 203 sessions were delivered in-person.
New sample size calculations were made, and the sample size reduced from 750 to 450 participants.	The sample size had to be reduced to make it possible to complete the trial due to increased workload for the NAV.	Not possible to compare the MI and SVAI interventions head-to-head due to reduced power.
Sensitivity analysis to test if the COVID-19 pandemic moderated the effectiveness of the MI or SVAI compared to UC.	The sensitivity analysis was conducted to investigate if the COVID-19 pandemic affected the trial results.	An exploratory analysis was conducted.

MI: motivational interviewing
SVAI: stratified vocational advice intervention

Appendix 4: BASELINE CHARACTERISTICS OF PARTICIPANTS IN THE MI-NAV TRIAL, ELIGIBLE INDIVIDUALS IN THE RECRUITMENT AREA AND IN THE WHOLE OF NORWAY

**Registry data**	**MI-NAV trial**	**Recruitment area ¤**	**Norway ¤**
	n = 514	*n* = 6329	*n = 140259*
Women, n (%)	293 (57)	3334 (53)	75412 (54)
Age, mean (SD)	48 (10)	47 (12)	46 (12)
Occupations, *n* (%)			
Legislator, senior officials, managers	9 (2)	448 (7)	9175 (7)
Professionals	94 (18)	918 (15)	22438 (16)
Technicians, associate professionals	30 (6)	653 (10)	14872 (11)
Clerks	41 (8)	395 (6)	8806 (6)
Service, shop and market sales workers	175 (34)	1835 (29)	39839 (28)
Agricultural, forestry and fishery workers	6 (1)	51 (1)	1474 (1)
Craft and related trade workers	63 (12)	847 (13)	17542 (13)
Plant and machine operators, assemblers	64 (13)	606 (10)	14108 (10)
Elementary occupations	31 (6)	565 (9)	11694 (8)
Armed forces and unspecified	1 (0.2)	11 (0.2)	311 (0.2)
Work, *n* (%)			
Full time: 100%	120 (69)	3788 (60)	87209 (62)
Part time: 50-99%	39 (22)	1339 (21)	27786 (20)
Part time: <50%	15 (9)	1202 (19)	25264 (18)

¤ Persons on sick leave for seven consecutive weeks for more than half of their contracted work hours due to a musculoskeletal disorder during the recruitment period of the MI-NAV Study: 5th of April 2019 to 14th of October 2020
n: number
SD: standard deviation

Aanesen F, *et al. Occup Environ Med* 2022; 80:42–50. doi: 10.1136/oemed-2022-108637
